# Transport of polymer-coated metal–organic framework nanoparticles in porous media

**DOI:** 10.1038/s41598-022-18264-y

**Published:** 2022-08-17

**Authors:** Satish K. Nune, Quin R. S. Miller, H. Todd Schaef, Tengyue Jian, Miao Song, Dongsheng Li, Vaithiyalingam Shuttanandan, B. Peter McGrail

**Affiliations:** 1 Energy and Environment Directorate, Pacific Northwest National Laboratory, Richland, WA 99354 USA; 2 Physical and Computational Sciences Directorate, Pacific Northwest National Laboratory, Richland, WA 99354 USA; 3 Environmental Molecular Sciences Laboratory, Pacific Northwest National Laboratory, Richland, WA 99352 USA

**Keywords:** Environmental sciences, Energy science and technology, Materials science, Nanoscience and technology

## Abstract

Injecting fluids into deep underground geologic structures is a critical component to development of long-term strategies for managing greenhouse gas emissions and facilitating energy extraction operations. Recently, we reported that metal–organic frameworks are low-frequency, absorptive-acoustic metamaterial that may be injected into the subsurface to enhance geophysical monitoring tools used to track fluids and map complex structures. A key requirement for this nanotechnology deployment is transportability through porous geologic media without being retained by mineral-fluid interfaces. We used flow-through column studies to estimate transport and retention properties of five different polymer-coated MIL-101(Cr) nanoparticles (NP) in siliceous porous media. When negatively charged polystyrene sulfonate coated nanoparticles (NP-PSS-70K) were transported in 1 M NaCl, only about 8.4% of nanoparticles were retained in the column. Nanoparticles coated with polyethylenimine (NP-PD1) exhibited significant retention (> 50%), emphasizing the importance of complex nanoparticle-fluid-rock interactions for successful use of nanofluid technologies in the subsurface. Nanoparticle transport experiments revealed that nanoparticle surface characteristics play a critical role in nanoparticle colloidal stability and as well the transport.

## Introduction

Nanoscale materials in the form of colloidal nanoparticles (nanofluids) are receiving increased attention for industrial and subsurface applications, including geologic carbon storage monitoring^1^, critical material extraction^2^, enhanced heat transport^3^, and hydrocarbon recovery^4^. At the same time, there is increased recognition of key roles that natural and anthropogenic nanomaterials play in Earth systems, although significant knowledge gaps with regards to nanoparticle fate and transport remain^5^. Unique properties such as extremely high surface area, diverse structures, transportability, and tunable surface functionalization make nanoparticles an attractive candidate for subsurface applications, and these properties are exemplified by metal–organic framework (MOF) materials. The small metal oxide clusters of these engineered porous materials are connected to organic linkers to produce hierarchically diverse topologies with a plethora of applications, including gas storage, separations, and catalysis^6^. Considerable attention has been devoted to developing synthesis methods for nanosized MOFs because of their promise in enhanced catalytic, sensing, and adsorption properties relative to bulk MOF forms^7,8^.

We recently demonstrated that MOFs are low-frequency, absorptive-acoustic metamaterials that exhibit anomalous sound transmission loss and tunable resonances from 100 to 1250 Hz^9^. These emergent low-frequency properties make MOFs desirable for sound-attenuating applications^10^, including for use as a geophysical contrast agent. We also have demonstrated that rocks saturated with MIL-101(Cr) (NP)^11^ nanofluids (~ 0.5 wt%) have distinct elastic and anelastic properties, resulting in decreased seismic wave velocities and amplitudes^12^. These attributes make injectable MOF nanoparticles a potentially disruptive technology for enabling geologic carbon storage and other subsurface energy storage/extraction endeavors. Our ongoing research involves using injectable colloidal MOF nanoparticles as geophysical contrast agents to help track fluids and delineate structures in the subsurface.

**Figure 1:**
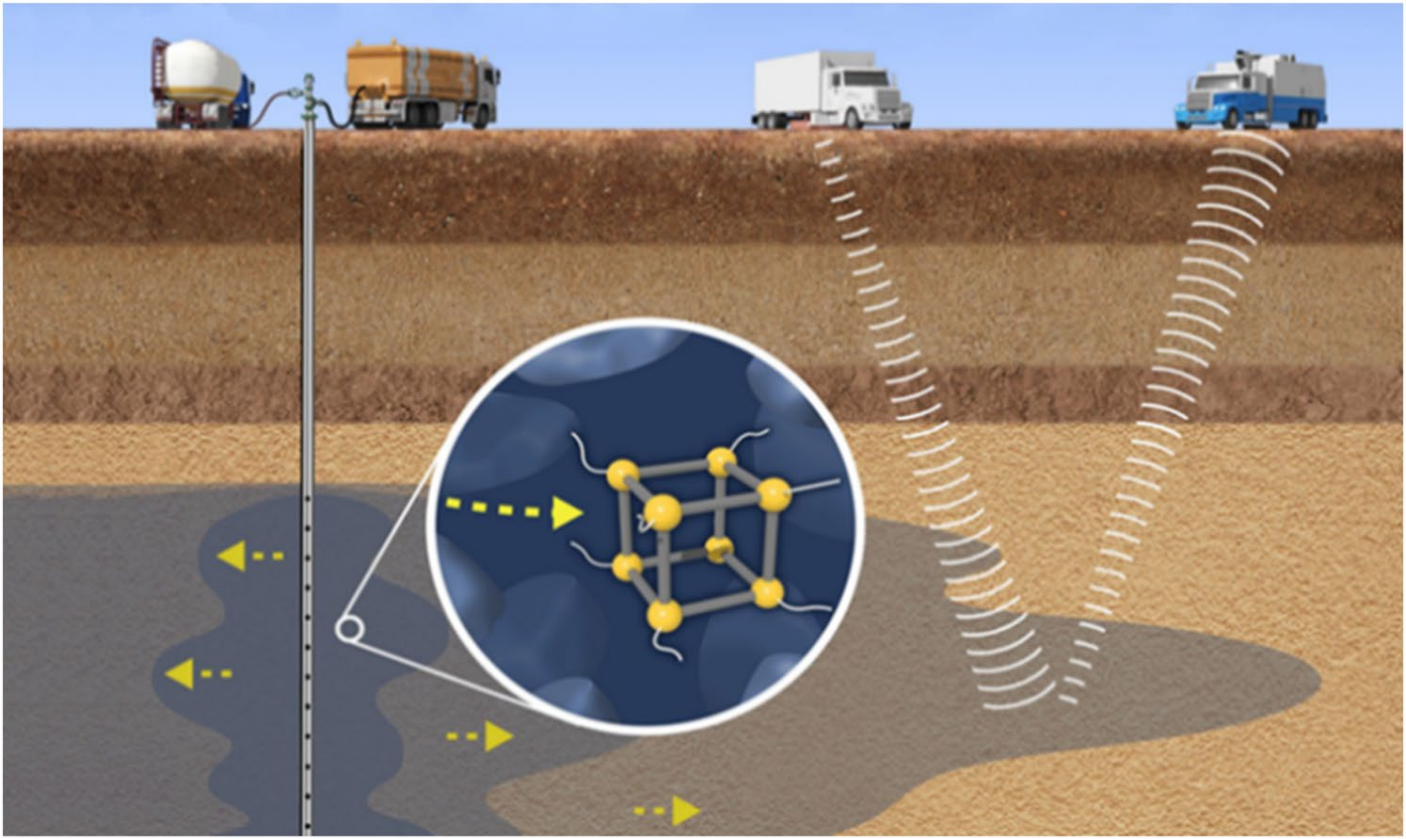
Illustration of low-frequency absorptive metal–organic frameworks acoustic nanomaterials injected into the subsurface to track fluids and map complex structures.

The goal of this present study is to examine how polymer coatings with different surface properties influence the ability of MOF nanoparticles to successfully traverse porous media relevant to geologic carbon storage reservoirs, a concept illustrated in Fig. 1.

A key requirement for a MOF nanofluid contrast agent is the ability of particles to be transported in porous geologic media without being immobilized in pores or agglomerated to other nanoparticles. To that end, we studied the effects of different polymer coatings on MOF nanoparticle transport and retention in siliceous porous geologic media, phenomena that not been evaluated previously. Polymer-coated MOF nanoparticle heterostructures were the focus of this work as we have recently demonstrated polymer coatings enhance colloidal stabilities of MOF nanoparticles, even when they are mixed with saline geothermal brines^13^.

## Experimental

### Materials

Accusand (Unimin Corporation), a natural geologic material with disseminated occurrence of clay minerals with opposing surface charges whose physical and chemical properties have been described by Schroth et al.^14^, was chosen for unconsolidated sand column experiments. Accusand (with no prior acid washes) was used as porous media in this study because of its relevant silica-rich composition, defined particle characteristics, and common use in nanoparticle transport studies^15,16^. The grain sizes of the Accusand was sieved to select the 210–841 µm size fraction (mesh 70–20), with 35% of the sand mass found in the 210–420 µm (mesh 20–40) range, and 65% of the mass occupying the 420–841 µm size range. Powder X-ray diffraction (PXRD) analyses indicated that only reflections assignable to quartz were present, matching International Centre for Diffraction Data powder diffraction file (PDF) #33-1161.

### Nanoparticle synthesis and polymer coating

Size-controlled synthesis^17–21^ of MOF particles was performed by using chemical modulators that compete with metal binding sites, thus dramatically reducing the number of active sites for crystal growth^8,17,19,20^. MIL-101(Cr) nanoparticles (NP) were prepared using a previously reported synthesis method^22,23^ in which terephthalic acid (139.6 mg, 0.82 mmol), modulator (4-methoxy benzoic acid; 5.1 mg, 0.033 mmol) and 25 mL of water were added to a fresh vial of Cr(NO_3_)_3_·9H_2_O (330 mg, 0.82 mmol). The heterogeneous suspension was mixed thoroughly followed by sonication for five minutes at room temperature and then heating to 180 °C for 4 h in a Teflon-lined autoclave. The reaction mixture then was cooled to room temperature and filtered with a 0.2 μm centrifuge filter to remove the unreacted/recrystallized terephthalic acid. After centrifuging the filtrate, the product was washed three times with water and methanol to obtain pure MIL-101(Cr) nanoparticles. As previously reported^22,23^, the as-synthesized MIL-101(Cr) nanofluid at an unadjusted pH of 5.5 was found to be colloidally stable and homogeneously dispersed for months, with high specific surface areas of 2917 m^2^/g.

Stable polymer-coated nanoparticle suspensions were prepared by mixing polymer solutions with aqueous-dispersed nanoparticles in an Orbital Shaker for ~ 4 h. The polymers used for MIL-101(Cr) nanoparticle (NP) coatings include poly(diallyldimethylammonium chloride) (PD1), polyvinyl pyrrolidone (PVP), polyethylenimine (PEI), and poly(sodium 4-styrenesulfonate) (PSS) (Fig. S1). Some of the polymers we surveyed (i.e., PSS, and PD1) were used previously in our laboratories for increasing colloidal stabilities of MOF nanoparticles against saline geothermal brines (Table S1). These polymer coatings were selected because of their differing surface charges, their hydrophobic behavior, and their effectiveness in coating MOF nanoparticles. PSS polymers with different average molecular weights of 70,000 and 200,000 were used to investigate size-dependent transport properties of polymer-coated nanoparticles, denoted as NP-PSS-70K and NP-PSS-200K. Excess polymer in the solution was removed by high-speed centrifugation, and the wet green pellet obtained was dispersed in water to obtain stable water dispersions of polymer coated MOFs for use in our nanoparticle transport study.

### Nanoparticle transport experiments

Transport of polymer-coated MIL-101(Cr) nanoparticles was evaluated by determining the quantity of MOF nanoparticles retained by the Accusand columns. Glass columns (10 cm diameter and 25 cm length) were used for studying nanoparticle transport and retention. The columns were wet packed with Accusand and moisturized at a water content of about 10% (*w*/*w*). To support column packing and retain the porous media, we used columns containing fritted discs (25–50 µm). The estimated flow rate was 0.30 cm/min, resulting in a residence time of 10.5 min. The relatively high flow rates and resultant short residence times were chosen to avoid any surface induced nanoparticle aggregation. Initially, uncoated nanoparticles were injected to understand the nanoparticle transport behavior. Nanoparticle effluent from the column was collected, and the concentration of nanoparticles was measured via ultraviolet–visible (UV–Vis) spectrometry. UV–Vis spectrometry is a relatively inexpensive and commonly applied method for determining colloidal nanoparticle concentrations^24^, and MIL-101(Cr) (NP) is resistant to acidic dissolution techniques needed for elemental analysis. Absorbance of the samples was measured over a wavelength range of 300–800 nm and the results presented are the average of at least two measurements. The absorbance of < 0.5 wt% colloidal MIL-101(Cr) water-based nanofluids (NP) are around ~ 560–580 nm, and the band in MIL-101(Cr) is due to π–π* transitions of ligands and the D–D spin-allowed transition of the Cr^3+^^25,26^.

## Effect of salinity on transport behavior of MOF nanoparticles

We conducted a series of nanoparticle transport experiments to evaluate the transport behavior of MOF nanoparticles with and without polymer coatings in saline environment. Transport experiments were conducted by wet packing the glass column with 50 g of Accusand with a pore volume of 14 mL. For estimating the effect of salinity on transport behavior of nanoparticles in Accusand, we used uncoated MOF nanoparticles and PSS-70K-coated MOF nanoparticles (Table S1). First, we flushed the packed column with about five pore volumes (PV) of water or diluted NaCl solution (1 M, 2 M, or 5 M). Once the column is saturated with effluent, we introduced 3.7 pore volumes of MOF nanoparticle suspension (in water or in diluted NaCl [1 M, 2 M or 5 M]) into the column and allowed them to flow through the column. We further flushed the column with another 1 pore volume (14 mL) of effluent (water or diluted NaCl [1 M, 2 M, or 5 M]). A total of 4.7 pore volumes of eluent is used in this transport study. The estimated flow rate was ~ 16 mL/min, resulting in a residence time of 4 min. Nanoparticle effluent from the column was collected, and the concentration of the nanoparticles was measured via ultraviolet–visible (UV–Vis) spectrometry.

We used a simple saturation method (porosity volume experiment) to estimate the pore volume of the Accusand. First, we mixed a known weight of Accusand and a known volume of water. A glass column was wet packed, and the volume of deionized water used at the saturation point in the column was noted. The pore volume of sand is determined as the difference between the total water volume before and after saturation. The porosity of the sand columns was 28% based on volumetric/gravimetric methods, not tracer testing. Our overall approach, including the number of pre-flushing pore volumes, porosity determination, and choice of Accusand material, is consistent with that used in a widely cited nanoparticle transport study^15^.

## Results and discussions

**Figure 2:**
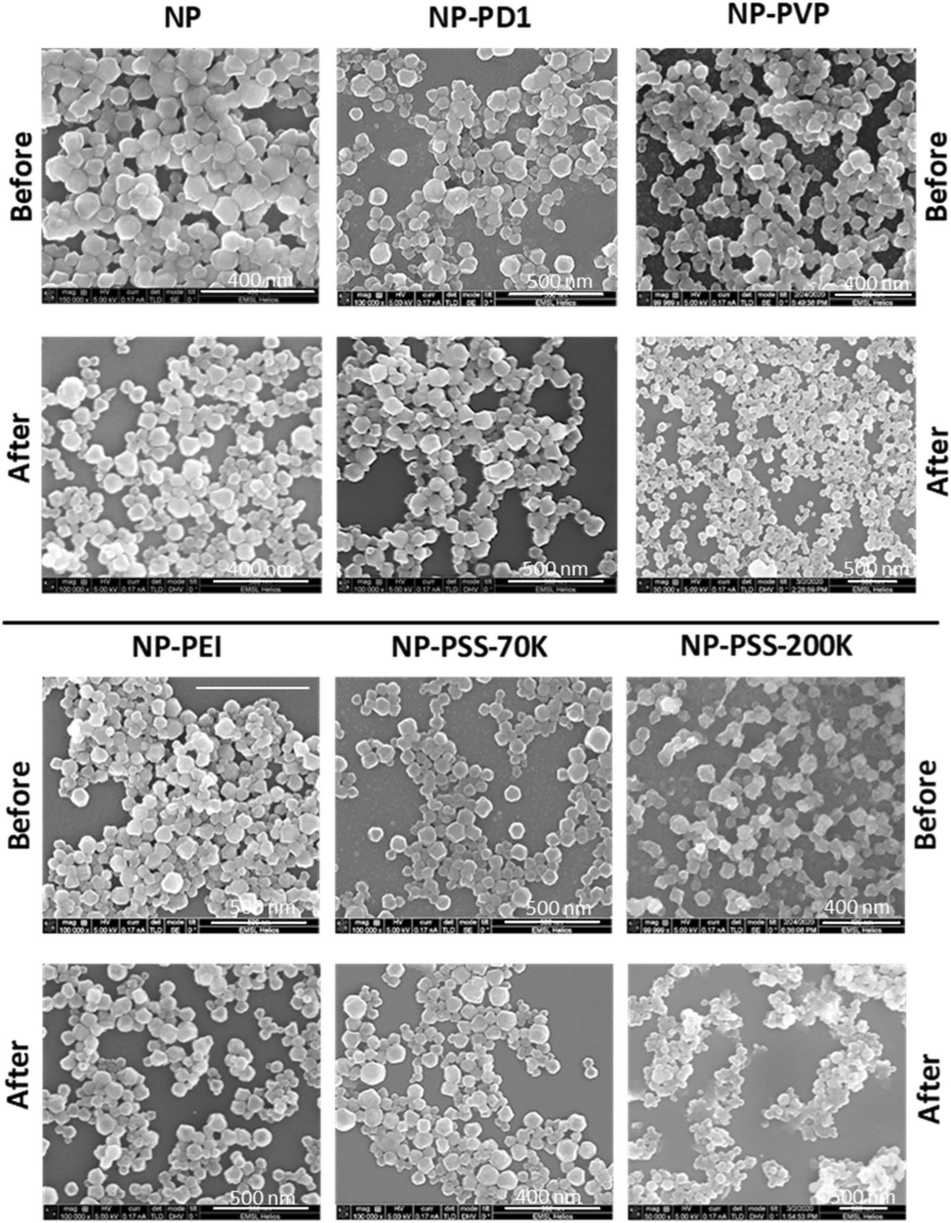
SEM images of MOF nanoparticles with different polymer coatings before and after Accusand column transport experiments.

Scanning electron microscopy (SEM) imaging characterization of our synthesized MOF nanoparticles revealed that the nearly spherical constituent nanoparticles were ~ 70–110 nm in diameter, and the overall morphological and size characteristics of the nanoparticles (NP-PD1, NP-PVP, NP-PEI and NP-PSS-70K) did not appear to change in response to polymer coating (Fig. 2). To understand the hydrophilic behavior of nanoparticles, we studied water adsorption and its potential impact on wettability alteration. Water adsorption isotherms (Fig. S2) revealed that the high sorption capacity (∼ 150 wt%) of uncoated MIL-101(Cr) nanoparticles (NP) was minimally affected by PD1 coatings. However, other samples exhibited decreased water loading at 60% relative humidity, including ~ 15% less uptake by NP-PSS-70K and ~ 60% less for both NP-PVP and NP-PEI. These results demonstrate that polymers may reduce access to MOF pore networks, and the polymers themselves may even partially occupy the pore volume. However, this polymer-coated MOF arrangement should not negatively impact their utility as acoustic-contrast agents, as synthesized MOFs (unactivated) still interact with acoustic waves^9^, and the polymers may even increase low-frequency attenuation, similar to the behavior of long-chain hydrocarbons^27^. We performed thermogravimetric analysis (TGA) of samples derived from soaking Accusand with polymer coated nanoparticles to gain insights on the amount of polymer coated nanoparticles adsorbed on Accusand (Fig. S3). TGA results revealed that MOF coated with a higher molecular weight polymer resulted in large percentage mass loss during TGA experiments (Fig. S3). TGA experiments confirmed that the percentage of mass loss appear to be more directly related to the polymer molecular weight than the type of polymer used for nanoparticle coating.

To further characterize the chemistry at the Accusand surface and polymer coated nanoparticles, we used X-ray photoelectron spectroscopy (XPS) to examine samples derived from soaking Accusand with polymer coated nanoparticles. XPS spectra of Accusand confirmed the presence of Si, O_2_, C, and a small amount of Nitrogen indicating that there is some charge heterogeneity on the Accusand surface. The presence of surface-associated nitrogen was observed in all the Accusand treated samples. The deconvoluted N 1* s* peaks at 401.8 and 404.1 eV are assigned to quaternary nitrogen bonded to silicon and free nitrogen on polymer, respectively (Fig. S4). The deconvoluted high-resolution XPS Cl 2*p* spectrum of an AS-NP-PD1 sample (Fig. S5) indicates the presence of polydadmac-Cl, Cl–N–Cr, Cl–N–Si species on the surface. The deconvoluted high-resolution XPS S 2*p* spectrum of an AS-NP-PSS-70K sample (Fig. S5) indicates the presence of Si–S, C–S, sulfite (C–SO_3_^2−^) species on the surface. XPS measurements corroborated the surface charge heterogeneities in Accusand that would have implications on nanoparticle transport/retention. Although it is not direct evidence of the stability and retention of nanoparticles, this information offers insights into surface characteristics that play a role in transport behavior of nanoparticles in porous media.

**Figure 3:**
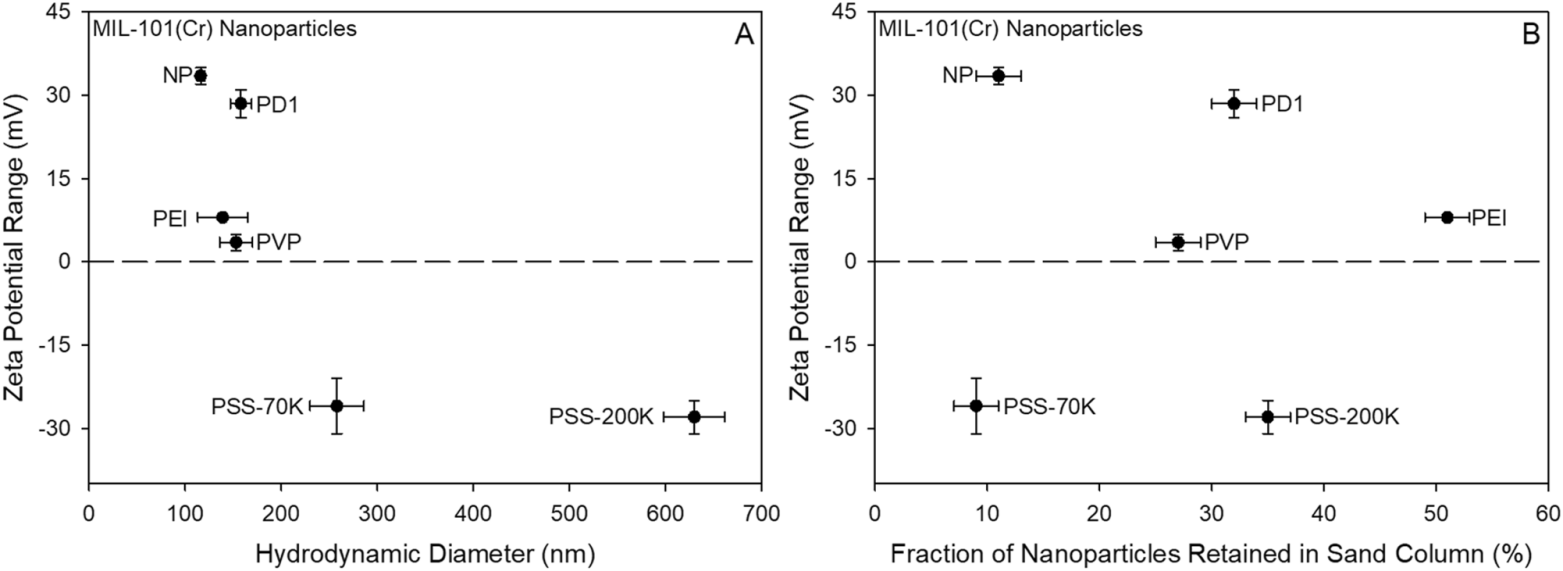
(**A**) Zeta potential ranges and hydrodynamic radii of synthesized MIL-101(Cr) MOF nanoparticles and polymer-coated MOF nanoparticles at an unadjusted pH of 5.5. (**B**) Results of flow-through transport experiments, showing how nanoparticle retention by Accusand was influenced by the polymer coating and electrokinetic.

We have recently conducted a detailed study on colloidal stability of various polymer-coated MOF nanoparticles in the presence of hydrochloric acid (HCl) and geothermal brines at different temperatures (US 2020/0384480 A1). Our studies revealed that out of five polymers tested, PSS improves the dispersion stability of MOF nanoparticles against geothermal brine by providing electrostatic repulsion between particles as characterized by the zeta potential (ζ)^28,29^. A summary on the stability of polymer coated MOF nanoparticle suspensions against brines using dynamic light scattering (DLS) measurements is provided in Table S1. As expected, the addition of polymer coatings altered the hydrodynamic and electrokinetic properties of the nanoparticles. The DLS showed that the hydrodynamic diameter of NP at an unadjusted pH of 5.5 was 116 nm (Table S2), but hydrodynamic diameters increased up to 632 nm for NP-PSS-200K, for a general trend of NP < PEI ≈ PD1 ≈ PVP < PSS (Fig. 3a).

The addition of PD1, PEI, and PVP polymer coatings decreased the zeta potential of the nanofluids relative to the ~  + 35 mV NP nanofluid (Table S2, Fig. 3a), making the polymer-coated nanoparticles more likely to adsorb and accumulate onto Accusand surface. The zeta potentials of PSS-coated samples were around − 27 mV, confirming that the particles have net negative charges, and the low absolute value indicates that the PSS-coated samples are as colloidally stable as the uncoated MIL-101(Cr) in distilled water. Overall, the ability to engineer novel nanoparticles that exhibit low frequency absorption with varying surface charges (surface as well the pores) is unique to MOF nanoparticles, as this allows the nanofluid injectate to be tailored to the subsurface reservoir composition, including for lithologies with negative (sandstone), positive (carbonates), or highly heterogenous surface charges (mudstones or clay-rich sandstones).

The most novel aspect of this present investigation is the transport behavior of MOFs in environmentally relevant porous media. To evaluate transport of MOF nanoparticles through porous media, we conducted flow-through column tests in which nanofluids passed through glass columns (10 cm diameter and 25 cm length) initially filled with water-saturated (10% w/w) quartz sand. Nanofluids had a linear flow rate of ~ 0.30 cm/min and a subsequent 10.5-min residence time in the column. Once the column is saturated with effluent, we used a total of 4.7 pore volumes of effluent in all the transport experiments (3.7 pore volumes of MOF nanoparticles [0.5 wt%] in water and flushed with 1 pore volume of water). As the focus of this paper is to investigate the five different nanoparticles coated with different polymers that differ in surface properties, a study outlining the influence of injected pore volume on transport/retention of nanoparticles with different polymer coatings injected pore volume was not done. Accusand^14^ was used as a model geo-substrate for column packing because of its well-defined silica grain size distribution, consistent morphology, relevant mineralogy for sandstone reservoirs^1,12^, and common use in nanoparticle transport studies^15,16^. We determined the percentage of nanoparticles transported by measuring nanoparticle concentrations in the effluent via the UV–Vis method and compared the results with the concentration of pre-injected nanoparticles.

Above a pH of ~ 2.5, silica sand has a net negative surface charge^30,31^, so it was expected that negatively-charged PSS-coated nanoparticles (Table S2) would most effectively be transported through the columns due to net electrostatic repulsion and subsequent unfavorable conditions for attachment. Indeed, only ~ 9% of NP-PSS-70K was retained in the core during the flow-through experiment (Fig. 3b, Table S2). The large hydrodynamic diameter of NP-PSS-200K relative to all other nanoparticles (Fig. 3a) was likely responsible for the > 3 × increase in retention relative to the NP-PSS-70K sample (Fig. 3b), especially considering that the two PSS-based nanofluids have nearly identical zeta potentials (Table S2, Fig. 3). Additionally, SEM imaging (Fig. 2) of nanoparticles transported through the column suggested that the NP-PSS-200K particles were more agglomerated after transport through the column, a characteristic that would not promote efficient transport. The ≥ 25% retention of the polymer-coated (PEI, PD1, PVP) nanoparticles with positive surface potentials was consistent with the hypothesis that electrostatic attraction predominantly drives particle retention. Of the three nanofluids, NP-PEI was the least effective in terms of transportability, likely due to a combination of low zeta potential of + 8 and its tertiary amine functional group, which has a strong affinity for negatively-charged surfaces (Table S2). SEM imaging of particles collected from the column effluent clarified that no observable changes in the size and/or morphology of individual nanoparticles were induced by transport through porous media (Fig. 2).

Finally, although the uncoated MIL-101(Cr) nanofluid has a net positive charge (Fig. 3a), only 11% of the particles were retained in the Accusand column. As we have previously injected this polymer-free nanofluid through a sandstone rock core^11^, this result was not surprising, although we did not anticipate that the mobility of nanoparticles in siliceous porous media would be on par with that of NP-PSS-70K. These results corroborate that a combination of factors such as size, surface charges, ionic strength, nature of stationary porous media, and nature of flow rate controls the transport of polymer coated MIL-101(Cr) nanoparticles^32^. In fact, smaller sizes would not necessarily exhibit the least retention, and in some cases, it would be in fact expected to show most retention^33,34^. Additionally, nanoscale surface charge heterogeneities in Accusand have been implicated for causing anomalous nanoparticle (de)sorption behavior^15,33^. Loss of 9% of NP-PSS-70K during transport is a reasonable loss along a 25 cm transport distance. The work presented in this paper highlights the importance of using a polymer coating with a suitable molecular weight and functional group density and type of MOF nanoparticles, its size and surface charges. Although there is some loss, the results are highly instructive in terms of down-selecting ideal polymer compositions for nano-MOF coatings.

**Figure 4:**
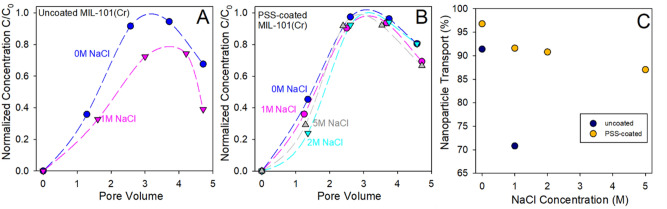
(**A**) Transport of uncoated nanoparticles (NP) in water and 1 M NaCl. (**B**) Transport of polymer-coated MOF nanoparticles (NP-PSS-70K) in water, 1, 2, and 5 M NaCl. (**C**) Comparison of nanoparticle transport for different salinity and particle type.

Nanofluid stability under different solution conditions (e.g., pH and salinity) is an active area of research because the high salt concentrations can reduce the electrostatic repulsion leading to flocculation behavior. We further investigated the effect of salinity on transport behavior of uncoated and polymer coated MOF nanoparticles in Accusand. Figure 4 illustrates the transport behavior of polymer coated nanoparticles with and without ionic strength solutions (0, 1, 2, and 5 M NaCl). The normalized effluent concentrations were plotted versus the number of pore volumes passed through the Accusand column. From Fig. 4a and Table S3 it is evident that there is significant retention (~ 29%) of nanoparticles observed when the transport of uncoated MIL-101(Cr) nanoparticles (NP) were conducted using 1 M NaCl solutions.

In contrast, only about 9% retention was observed when uncoated MIL-101(Cr) nanoparticles were transported using water (Fig. 4b). Interestingly, when negatively charged PSS-coated nanoparticles were transported in 1 M NaCl, only about 8.4% nanoparticles were retained in the column. When the ionic strength of the medium was increased to 2 M NaCl, we observed only about 9% NP-PSS-70K retention in the core during the flow-through experiment. When the ionic strength of the medium was further increased to 5 M NaCl, we still observed only 13% of NP-PSS-70K retained. Transport experiments confirmed that polymer poly(sodium 4-styrenesulfonate; PSS), an adsorbing polymer not only enhances the colloidal stability of nMIL-101 nanoparticles (NP) at room temperature but also plays critical role in the transport of nanoparticles in porous siliceous media (Fig. 4c). Although the presence of high concentration of ions at high salt concentrations can enhance the interactions of the ionic groups on polymers, the sodium salt of polystyrene sulfonate polymer on MOF nanoparticles provides electrostatic repulsion, as characterized by the zeta potential (ζ)^28,29^. When we conducted transport experiments with solutions with different salinities, no transient solution salinities occurred. When the solution salinity was reduced to near zero, we did not observed any clay mobilization because Accusand was used in our studies as the porous media because of its relevant silica-rich composition. These results reaffirm the role of nanoparticle surface characteristics on colloidal stability and their transport in environmentally relevant porous media.

## Conclusion

In summary, we have demonstrated that the polymer coating on MOF nanoparticles will have significant impact on their transport in porous media. To the best of our knowledge, these results represent the first report on the transport properties of polymer-coated MOF-nanoparticles in environmentally relevant porous media. Of the polymer-coated nanoparticles studied, NP-PSS-70K was best able to migrate through the sand columns without being retained, and PEI-coated nanoparticles exhibited the worst performance, as over 50% of the particles did not pass through the Accusand. The PEI results especially emphasize that complex nanoparticle-fluid-rock interactions must be understood for successful use of nanofluid technologies in the subsurface. Surface adsorption of nanoparticles onto mineral interfaces must be minimized for successful injection and operation of novel seismic contrast agents, and retention is dominantly controlled by solution ionic strength, nanoparticle surface potentials, exposed functional groups, and effective size of colloids. To that end, we have designed, synthesized, and tested different polymer-coated MOF nanofluids with varying surface charges and functionalities that may be deployed in a variety of subsurface reservoirs with varying formation fluid chemistry and rock types.

## Supplementary Information

Supplementary Information.
