# Supplementary material for: Transport of polymer-coated metal–organic framework nanoparticles in porous media
Source: Sci Rep. 2022 Aug 17;12:13962. doi: 10.1038/s41598-022-18264-y (PMC9385709; doi:10.1038/s41598-022-18264-y)
Supplement: Supplementary file 1 — Supplementary Information. [file 41598_2022_18264_MOESM1_ESM.docx]

**Supporting Information for**

**Transport of Polymer-Coated Metal-Organic Framework Nanoparticles in Porous Media**

Satish K. Nune,^1*^ Quin R. S Miller,^2^ H. Todd Schaef,^2^ Tengyue Jian,^2^ Miao Song,^2^ Dongsheng Li,^2^ Vaithiyalingam Shutthanandan^3^ and B. Peter McGrail ^a^

^1^Energy & Environment Directorate, Pacific Northwest National Laboratory, Richland, WA 99354, USA

^2^Physical and Computational Science Directorate, Pacific Northwest National Laboratory, Richland, WA 99354, USA

^3^Environmental Molecular Sciences Laboratory, Pacific Northwest National Laboratory, Richland, WA 99352, USA

*Corresponding Author Email: [satish.nune@pnnl.gov](mailto:satish.nune@pnnl.gov)

**Characterization Methods**

**Scanning Electron Microscopy**

Aqueous uncoated and polymer coated nanoparticles were sonicated for a few minutes and then ~200 µl of the solution was drop cast on a Si wafer and dried in air. Scanning electron microscopy was performed with a JEOL 7600 FEG SEM at low 3-5 kV accelerating voltages in both secondary and backscatter modes.

**Zeta Potential and Dynamic Light Scattering**

The zeta potential (ζ-potential) values of the MIL-101(Cr) nanoparticles were measured by using Brookhaven NanoZeta potential (ZetaPALS) at room temperature (~25 °C). The final ζ-potential values for all polymer-coated nanoparticles were obtained by averaging three measurements. The pH of the freshly prepared uncoated nanoparticles was measured by Horiba compact pH meter. Dynamic light scattering (DLS) measurements performed on a Brookhaven Instruments Corporation Zeta Phase Analysis Light Scattering analyzer were used to determine the hydrodynamic diameters of MOF nanoparticles.

**Ultraviolet-Visible Spectrometry**

Ultraviolet-visible spectrometry (UV-Vis) measurements were made on recovered effluent from the flow-through sand column experiments. UV-vis was performed on a Ultrospec 2100 pro using 1 cm path length quartz cells. The UV–vis absorbance values were measured at wavelengths from 350 nm to 700 nm and water was used as the blank sample. To measure the nanoparticle retention profile, the concentration of the nanoparticles coming out of the column was measured using UV-Vis spectroscopy. The absorbance of the samples was measured over a wavelength range of 300–800 nm and the results presented are the average of at least two measurements. Colloidal nanoparticles (~ 0.5 wt% in water) have absorbances at ~560-580 nm. The unique on ultraviolet-visible (UV-Vis) spectra can be utilized to calculate nanoparticle concentrations. Concentrations of colloidal MOF nanoparticles were determined with the Beer-Lambert law that uses unique absorption peaks of nanoparticles in ultraviolet-visible (UV-Vis) spectra:

*A* = *εbc* (Eq. 1)

where *A* is absorbance, ε is the molar extinction coefficient with unit of M^−1^ cm^−1^, *b* is the path length of the sample (cm), *c* is the concentration of nanoparticles in solution (M).

In order to make calibration curves, five different known concentrations of each nanoparticle were prepared to measure the UV-Vis absorbance. For each concentration of nanoparticle, the maximum absorbance was determined. Using these samples with known concentrations and measured absorbances, calibration curves were plotted to determine the linear absorbance-concentration relationships for each.

**SUPPLEMENTARY TABLES**

| **Sample No** | **Sample** | **Polymer Coating** | **Hydrodynamic Diameter (nm)** |
| --- | --- | --- | --- |
| 1 | NP | uncoated MIL-101(Cr) | 116.3 |
| 2 | NP-HCl | uncoated MIL-101(Cr)treated with hydrochloric acid | 2007 |
| 3 | NP-PD1 | poly(diallyldimethylammonium  chloride), 1 wt% | 158 |
| 4 | NP-PD1-HCl | poly(diallyldimethylammonium  chloride), 1 wt% | 552 |
| 5 | NP-PD1-1M NaCl -70 °C | poly(diallyldimethylammonium  chloride), 1 wt% | 180 |
| 6 | NP- PD1-1M NaCl-95 °C | poly(diallyldimethylammonium  chloride), 1 wt% | 190 |
| 7 | NP- PD1-Geothermal Brine-70 °C | poly(diallyldimethylammonium  chloride), 1 wt% | 5114 |
| 8 | NP- PD1-Geothermal Brine-95 °C | poly(diallyldimethylammonium  chloride), 1 wt% | 3308 |
| 9 | NP-PSS-70K -RT | poly(sodium 4-styrenesulfonate-70K, 2 wt% | 435 |
| 10 | NP-PSS-70K -95 °C | poly(sodium 4-styrenesulfonate-70K, 2 wt% | 294 |
| 11 | NP-PSS-70K-Geothermal Brine-RT | poly(sodium 4-styrenesulfonate-70K, 2 wt% | 477 |
| 12 | NP-PSS-70K-Geothermal Brine-95 °C | poly(sodium 4-styrenesulfonate-70K, 2 wt% | 510 |

**Table S1: Survey of stability of polymer coated MOF nanoparticle suspensions against brines using DLS measurements**

| **Nanofluid Sample Name** | **Polymer Coating** | **Molecular Weight of Polymer** | **Hydrodynamic Diameter (nm)†** | **Zeta Potential Range (mV)** | **Amount of Nanoparticle Retained in Accusand Column Experiments (%)‡** | **Nanofluid pH** |
| --- | --- | --- | --- | --- | --- | --- |
| NP | uncoated MIL-101(Cr) | n/a | 116 (4) ^13^ | +32 to +35 ^14^ | 11 | 5.97 |
| NP-PVP | poly(vinylpyrrolidinone) | 40000 | 153 (17) | +2 to +5 | 27 | 6.20 |
| NP-PD1 | poly(diallyldimethylammonium  chloride), 1 wt% polydadmac | 200,000-350,000 | 158 (11) | +26 to +30 | 32 | 4.73 |
| NP-PSS-70K | poly(sodium 4-styrenesulfonate-70 K, 2 wt% | 70,000 | 258 (28) | -21 to -31 | 9 | 6.60 |
| NP-PSS-200K | poly(sodium 4-styrenesulfonate-200 K, 2 wt% | 200,000 | 630 (32) | -25 to -31 | 35 | 6.89 |
| NP-PEI | Polyethylenimine, 2 wt% | 10000 | 139 (26) | +8 | 51 | 7.20 |

**Table S2**. List of polymer-coated MOF nanoparticles with dynamic light scattering (hydrodynamic diameter), and zeta potential results.

| **Nanofluid Sample Name** | **Polymer Coating** | **NaCl Concentration (M)** | **Initial Nanofluid Concentration (wt%)** | **Amount of Nanoparticle Retained in Accusand Column Experiments (%)‡** |
| --- | --- | --- | --- | --- |
| NP | n/a, uncoated MIL-101(Cr) | 0 | 0.2596 | 8.6 |
| NP | n/a, uncoated MIL-101(Cr) | 1 | 0.2596 | 29.2 |
| NP-PSS-70K | poly(sodium 4-styrenesulfonate-70K, 2 wt% | 0 | 0.3688 | 3.2 |
| NP-PSS-70K | poly(sodium 4-styrenesulfonate-70K, 2 wt% | 1 | 0.4253 | 8.4 |
| NP-PSS-70K | poly(sodium 4-styrenesulfonate-70K, 2 wt% | 2 | 0.2970 | 9.2 |
| NP-PSS-70K | poly(sodium 4-styrenesulfonate-70K, 2 wt% | 5 | 0.2596 | 13.0 |
| Abbreviations: n/a, not applicable  ‡ Estimated uncertainty is ±2% | | | | |

**Table S3**. Results for MOF nanofluid transport as a function of NaCl concentration and coated vs. PSS-coated.

**SUPPLEMENTARY FIGURES**


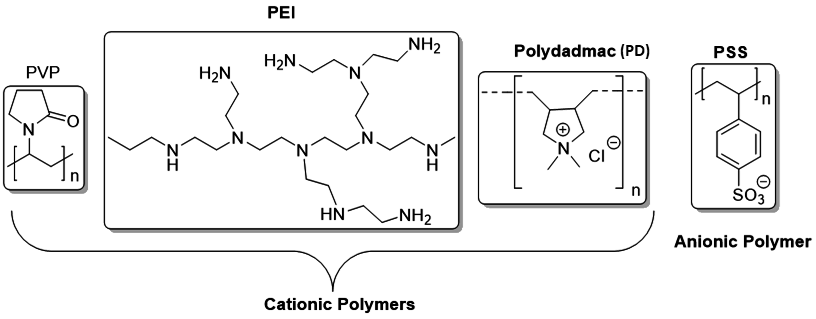


polyvinyl pyrrolidone (PVP), polyethylenimine (PEI), poly(diallyldimethylammonium chloride) (PD1), and poly(sodium 4-styrenesulfonate) (PSS)

**Figure S1**: List of polymers studied for nanoparticle coating


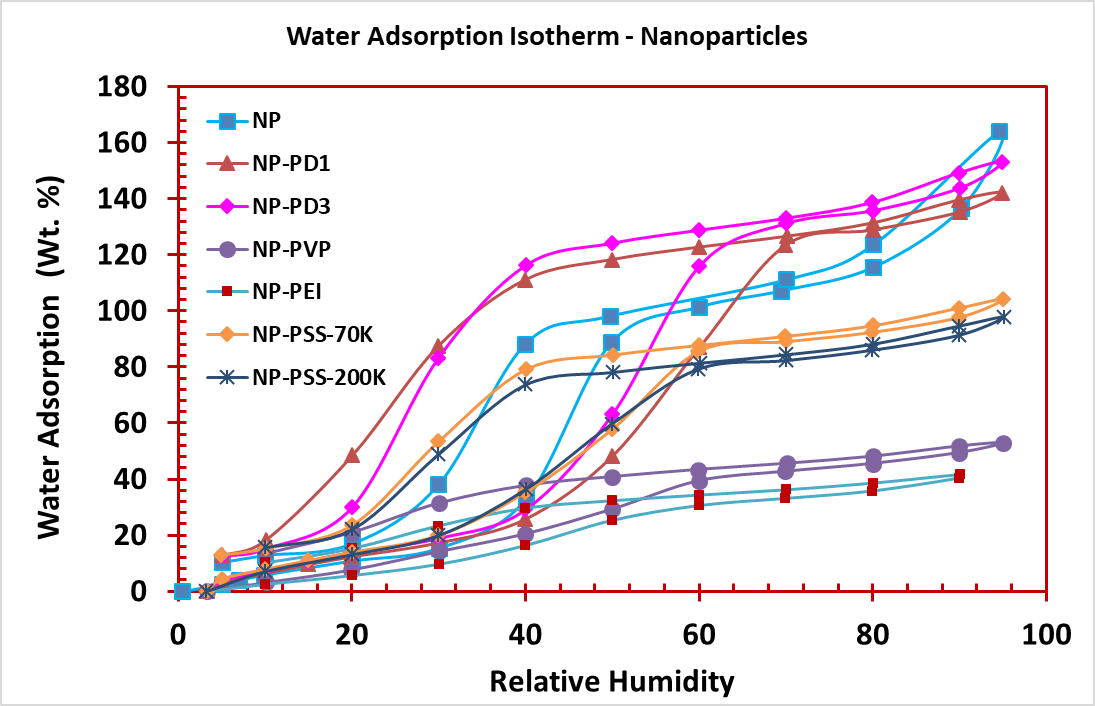


**Figure. S2:** Water adsorption isotherms at 25 °C for uncoated **NP** and polymer-coated nanoparticles (**Table S1**).


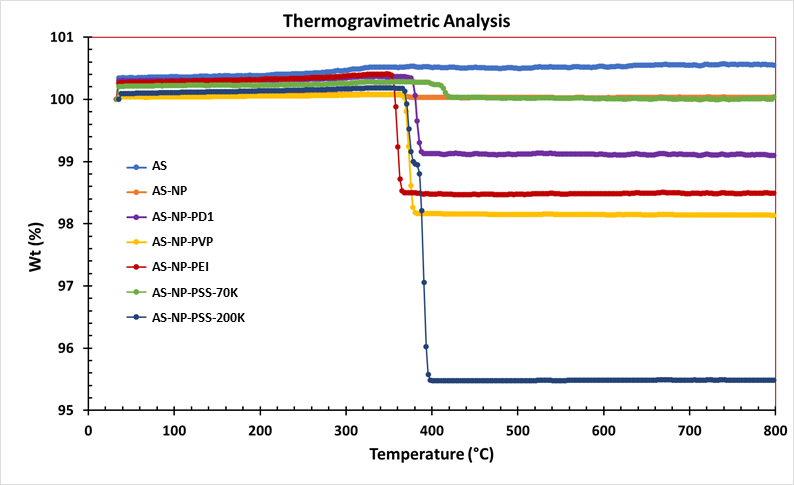


**Figure S3**: Thermogravimetric analysis (TGA) of Accusand and Accusand treated with polymer-coated MOF nanoparticles.

To understand MOF nanoparticles transport behavior fully, understanding the interaction between Accusand and uncoated/ polymer coated nanoparticles is very important. We performed thermogravimetric analysis (TGA) of samples derived from soaking Accusand with polymer coated nanoparticles to gain insights on the amount of polymer coated nanoparticles on Accusand. The percentage (%) mass loss of all the samples studied is illustrated in **Figure S3**. The TGA of the Accusand showed no mass loss between 40-800 °C (**Figure S3**), and TGA of Accusand treated with high molecular weight PSS-200K (**AS-NP-PSS-200K**) showed higher mass loss (~4.5 wt%) compared to all other samples tested (**Figure S3**). The mass loss occurred at around 350-400 ^o^C indicated the decomposition of polymer and MOF. TGA analysis of Accusand treated with uncoated nanoparticles (**NP**) showed slight weight loss (~1 wt %). Interestingly, TGA of **AS-NP-PSS-70K** revealed that it has only about <1 wt % between 40 and 800 ^0^C indicating only small amount of polymer coated nanoparticles were adsorbed. Zeta potential measurements of **NP-PSS-70K** (-21 to -31 mV) corroborate the poor adsorption of **NP-PSS-70K** nanoparticles on to Accusand. However, TGA analysis confirmed that the **NP-PSS-200K** has higher adsorption on Accusand followed by **NP-PVP** (2 wt%). TGA results revealed that MOF coated with higher molecular weight polymer resulted in large % mass loss during TGA experiments (**Figure S3**). Based on the molecular weight of the polymers, the expected % mass loss is PSS-200>PD#>PVP>PEI. Sure enough, we observed the same in TGA. TGA experiments confirmed that the percentage of loss appear to be more directly related to the polymer molecular weight than the amount of nanoparticles adsorbed.

**Figure S4**: Deconvoluted high-resolution X‑ray photoelectron spectroscopy (XPS) spectra of Accusand and Accusand treated with polymer-coated nanoparticles

**Figure S5**: Deconvoluted high-resolution X‑ray photoelectron spectroscopy (XPS) spectra of **AS-NP-PD1** and **AS-NP-PSS-70K**
